# Go-Game Image Recognition Based on Improved Pix2pix

**DOI:** 10.3390/jimaging9120273

**Published:** 2023-12-07

**Authors:** Yanxia Zheng, Xiyuan Qian

**Affiliations:** School of Mathematics, East China University of Science and Technology, Shanghai 200237, China; y30211280@mail.ecust.edu.cn

**Keywords:** pix2pix, image recognition, CCMA, DDC

## Abstract

Go is a game that can be won or lost based on the number of intersections surrounded by black or white pieces. The traditional method is a manual counting method, which is time-consuming and error-prone. In addition, the generalization of the current Go-image-recognition methods is poor, and accuracy needs to be further improved. To solve these problems, a Go-game image recognition based on an improved pix2pix was proposed. Firstly, a channel-coordinate mixed-attention (CCMA) mechanism was designed by combining channel attention and coordinate attention effectively; therefore, the model could learn the target feature information. Secondly, in order to obtain the long-distance contextual information, a deep dilated-convolution (DDC) module was proposed, which densely linked the dilated convolution with different dilated rates. The experimental results showed that compared with other existing Go-image-recognition methods, such as DenseNet, VGG-16, and Yolo v5, the proposed method could effectively improve the generalization ability and accuracy of a Go-image-recognition model, and the average accuracy rate was over 99.99%.

## 1. Introduction

Originating in China, Go is an important competitive game that is widely known. Go consists of a square board with black and white round pieces, and 19 vertical and horizontal lines on the board divide it into 361 intersections on which the black and white pieces land. During the game, two players take turns to place pieces and, at the end, the number of black and white pieces are added to the number of intersections surrounded by them, and the side with higher number is the winner. The most commonly used calculation method is the manual counting method, but the manual method is inefficient and prone to miscounting. At present, a machine-assisted method has been applied in many competitions, which has greatly improved the efficiency and accuracy, as compared to the manual method. Therefore, computer-assisted counting can be used, but the problem remains regarding how a computer can accurately identify images of a Go game, that is, correctly identify the black and white pieces and their positions.

The current Go-image-recognition methods are divided into two categories. One is the traditional image-processing methods based on OpenCV. The advantages of these methods are their simple model structures and small calculation amounts. However, the disadvantages are that most only consider the current information in the image, which is usually not robust to noise and thus unable to obtain satisfactory results in complex scenes, resulting in insufficient generalization. The other is deep-learning processing methods based on convolutional neural networks. The advantages of these methods are that the accuracy of the model increases. However, due to the need to use multiple models for checkerboard recognition and chess-piece recognition, the number of parameters in the model increases, and then the inference speed decreases.

To this end, we constructed a new Go-game-image dataset. Furthermore, we proposed a chess-image-recognition algorithm based on an improved pix2pix. In order to pay more attention to the local details of the input image, a channel-coordinate mixed-attention (CCMA) structure was proposed. At the same time, in order to better capture the semantic information based on a long-distance context, a new deep, extended convolution module (DDC) was proposed. With these two new modules added to the pix2pix, the experiment showed that the improved model could handle a scenario with a complex background while improving the accuracy and enhancing the robustness. In addition, the model recognized the whole image, which greatly sped up the inference speed.

## 2. Related Work

A lot of work has been complete in order to recognize Go game images by using traditional image-processing methods. Huang [1] proposed a chess recognition algorithm based on chain coding, which used vertex chain-coding technology to parse images into coding trees and then used computer-aided notation to judge the winners and losers. However, this method required manual input. Seewald [2] proposed an optimized SVM to identify chess spectrum images from video records of Go games under constrained conditions, but the recognition rate of this method was only 72.7%. Chang [3] proposed a Go-image-segmentation algorithm based on OpenCV, but it did not perform well on a board with a complex background. Gui [4] used image-processing technology to detect, locate, and segment chess pieces in a chess game involving board information extraction of the Go robot with an accuracy rate of 93.3%, but the problem of illumination influence was not solved. On this basis, Zhao [5] used an MLP model to overcome the influence of uneven illumination, and the recognition accuracy of the Go robot was more than 90%. The above methods involved simple structures and small amounts of calculation but had the problem of insufficient generalization.

With the development of deep learning, convolutional neural networks (CNN) have been widely used for image recognition. At present, there is much research on Go-game image recognition based on CNN. Czyzewski [6] proposed a digital chessboard-configuration algorithm by combining traditional algorithms with neural networks. Based on this algorithm, he designed a chessboard-recognition and chess-piece-detection method, and the accuracy rate of the chess-piece recognition was close to 95%. Quintana [7] proposed a functional framework called LiveChess2FEN that could digitize chess-game images in real time. After positioning the board, the system used a network model, such as DenseNet, to classify all the pieces, eventually achieving 92% accuracy. Neto [8] proposed a method for identifying synthetically generated chess images in Blender using a Python API, achieving 97% accuracy in chess-piece classification by fine-tuning the VGG-16 convolutional network. Zhuo [9] proposed a Go-image-recognition method based on Yolo v5 that could resist the influence of light reflection, but the model was more complex and required higher computational performance. The above methods had the advantages of better model accuracy and robustness. However, these methods required multiple models to identify the chessboard and the chess pieces successively, which thus increased the workload of dataset annotation and the number of parameters of the model.

Since Goodfellow [10] proposed generative adversarial nets (GANs), GANs have become a hotspot in the research. Since GAN do not require much labeling of training datasets, the pre-processing work is greatly simplified. However, since GANs are too free to be controllable, Mirza [11] proposed conditional generative adversarial nets (CGAN), which constrained the generation of the models by adding a condition y. Based on this, Isola [12] proposed pix2pix, which greatly improved the accuracy of generated images by introducing paired data labels. Lundine [13] compared various models, including pix2pix, when identifying pits in various seabed environments and obtained high accuracy and recall rates. Therefore, it was reasonable to consider using pix2pix as the basic model, which has achieved high accuracy in image recognition, and pix2pix directly recognizes an entire image, reducing the annotation work on the dataset.

## 3. Basic Model

**Figure 1:**
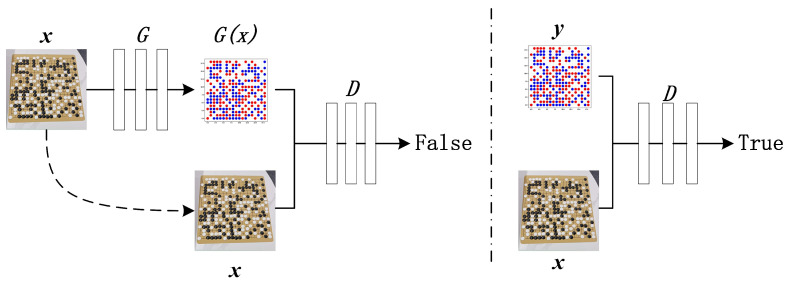
Pix2pix training principle.

The generative adversarial network (GAN) was composed of two parts: the generative model (G) and the discriminative model (D). The generator used random noise as input, and its training goal was to learn the distribution of real data in order to generate pseudo-data similar to real data. The goal of the discriminator training was to accurately distinguish between real data and fake data. The original GAN had no constraints on the generator, which increased the randomness of the data generation. CGAN adds a constraint condition *y* on the basis of the original GAN in order to make the network generate samples in a given direction. However, both GAN and CGAN use random noise as input, and although the generated data could learn the distribution of real data, it was not sufficiently related to the input data. Pix2pix was an improvement based on CGAN where the input was no longer random noise but, instead, sample data *x*. The implementation principle is shown in Figure 1.

**Figure 2:**
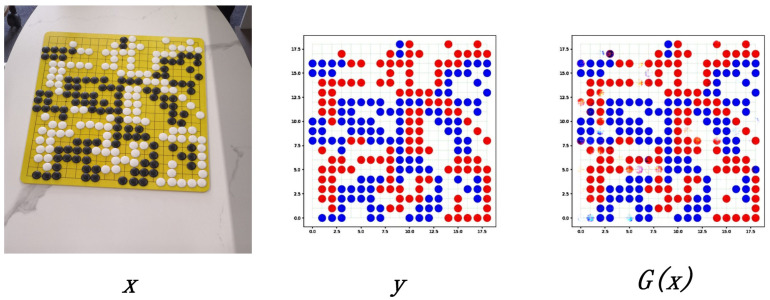
An example of pix2pix training.

Figure 2 shows an example of the pix2pix recognition. It was observed that for the original pix2pix, although the image *G(x)* generated by pix2pix was close to the label *y*, most chess pieces could be accurately identified, but a careful observer will find that there were many cases of poor chess-piece recognition, missing detection, and incorrect color identification of the chess pieces. This shows that the original pix2pix could not meet the demand, and it needed to be optimized to improve the accuracy of the model.

## 4. Improved Pix2pix

### 4.1. Generator

**Figure 3:**
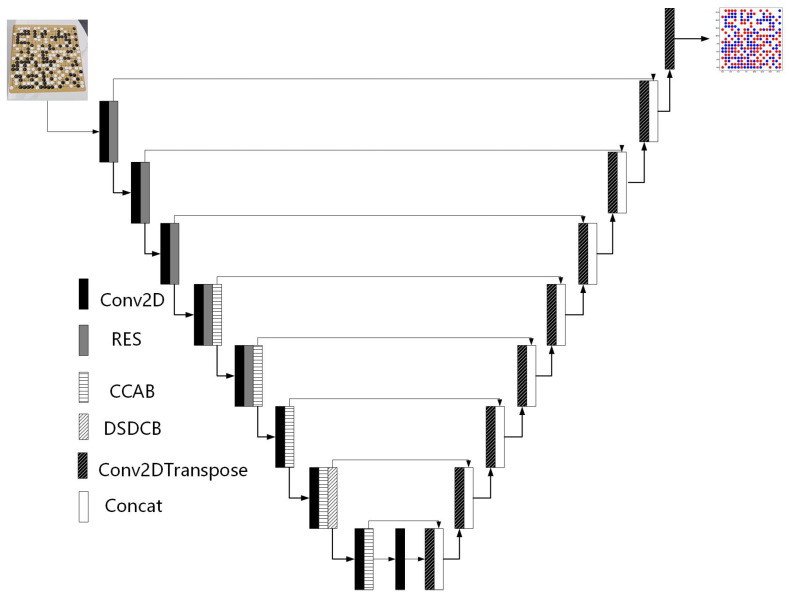
Generator network.

Pix2pix used the U-net [14] as a generator. For the generator, this study retained the U-Net structure in the original pix2pix, including down-sampling, up-sampling, and skip connection. The specific structure is shown in Figure 3. Although skip joins were used to fuse semantic information at each level, a large amount of information was still lost during down-sampling. During the task of image recognition of Go in this study, the model needed to learn more accurate pixel-level prediction. Therefore, CCMA and DDC modules were added into the down-sampling process of the U-net network. The former module enabled the model to learn the target feature information with increased attention, and the latter module enabled the model to learn the long-distance contextual information at different scales.

#### 4.1.1. Channel-Coordinate Mixed Attention (CCMA)

By changing the weights of different features in the image, the attention mechanism could make the model quickly focus on the key information in the image and improve the feature-expression ability of the model. Hu [15] proposed that the SENet network adapted the characteristic response of the calibration channel by explicitly modeling the interdependence between the channels. However, the SENet network only considered the channel information inside the image, ignoring the position information. Woo [16] proposed that the convolutional block attention module (CBAM) introduced positional information through global pooling in the channels, combining channel attention with spatial attention. However, this method could only obtain local position information, and the texture details of the generated image were missing. Hou [17] put forward the coordinate attention (CA) module, which extracted the attention-feature information in the X direction and the Y direction, so as to obtain the global position information of the input feature map. However, when using this method, the position information was embedded in each channel, so the relationship between the features of each channel was weakened.

**Figure 4:**
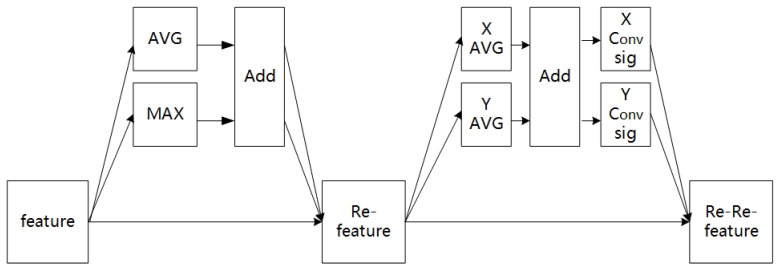
Channel-coordinate mixed-attention mechanism.

Based on the above attention methods, this study proposed the channel-coordinate mixed-attention (CCMA) mechanism. As shown in Figure 4, CCMA contained two independent sub-modules. In the first sub-module, the input feature map was firstly subjected to global maximum pooling (MAX) and global average pooling (AVG), and then the two output feature maps were added. Finally, the channel feature map (Re-feature) was obtained by weighting the previous features, one by one, through multiplication in order to complete the re-calibration of the original features in the channel dimension. In the second sub-module, the Re-feature underwent global average pooling (X-MAX) in the X direction and Y-AVG in the Y direction, and the two output feature maps were separated after the add-operation; then, the two coordinate-attention features were generated after a 1 × 1 convolution-and-activation operation. Finally, the feature re-calibration in the coordinate dimension was completed by multiplication, and the channel-coordinate feature map (Re-Re-feature) was obtained. The module combined channel attention with coordinate attention effectively by extracting channel- and coordinate-attention features successively. Not only was the cross-channel feature information captured, but also the position coordinate information of the features in each channel was considered, so that the model could obtain a better feature representation.

#### 4.1.2. Depthwise Dilated Convolution (DDC)

The dilated convolution was originally proposed to solve the problem of image segmentation, as it could increase the receptive field while keeping the size of the feature map unchanged, so as to retain most of the information in an image. Chen [18] proposed the atrous spatial pyramid pooling in DeepLab v2 to capture the contexts of images by parallel sampling the void-convolution at different sampling rates for given inputs. Dai [19] combined the densely connected convolutional network with a dilated convolution and proposed a densely dilated convolution block to capture a wide range of scale changes in the densest possible way. The dense connection performance was good, but the parameters exponentially increased.

**Figure 5:**
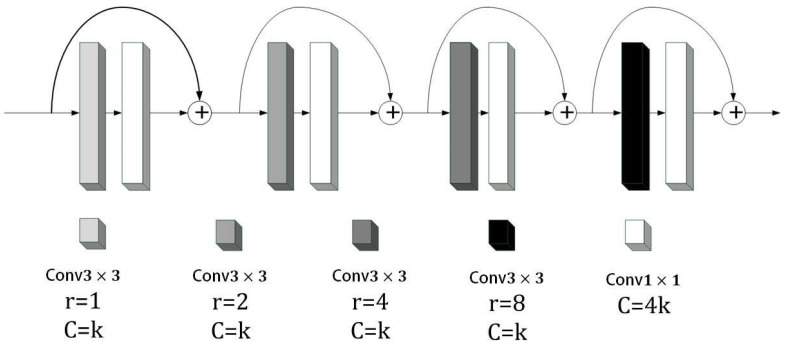
Depthwise dilated convolution module.

Based on the above methods, the depthwise dilated convolution (DDC) was proposed in this study. As shown in Figure 5, the dilated convolution was introduced to increase the model’s receptive field. Although the dilated convolution increased the receptive field without losing the size of the feature graph, it also introduced new problems. Since the dilated convolution kernel was spaced when scanning the image, this meant that not all pixels in the image were involved in the calculation. Therefore, four convolution layers with different dilation rates, 1, 2, 4, and 8, were used to ensure that all inputs participated in the calculation. In order to reduce the parameters of the model, Chollet [20] proposed depthwise separable convolutions, which greatly reduced the parameters of the convolution by replacing 2D convolution with channel-by-channel and point-by-point convolutions. Inspired by this, each dilated convolution layer in this study was changed to consist of a combination of a 3 × 3 channel-by-channel expanded convolution and a 1 × 1 point-by-point convolution. This setup preserved information from denser scales with fewer parameters. Considering that point-by-point convolution lacked the spatial correlation information, the four layers adopted a dense connection method to detect convolutional features on multiple scales and realized continuous information transfer, thus learning long-distance contextual information at different scales.

### 4.2. Discriminator

**Figure 6:**
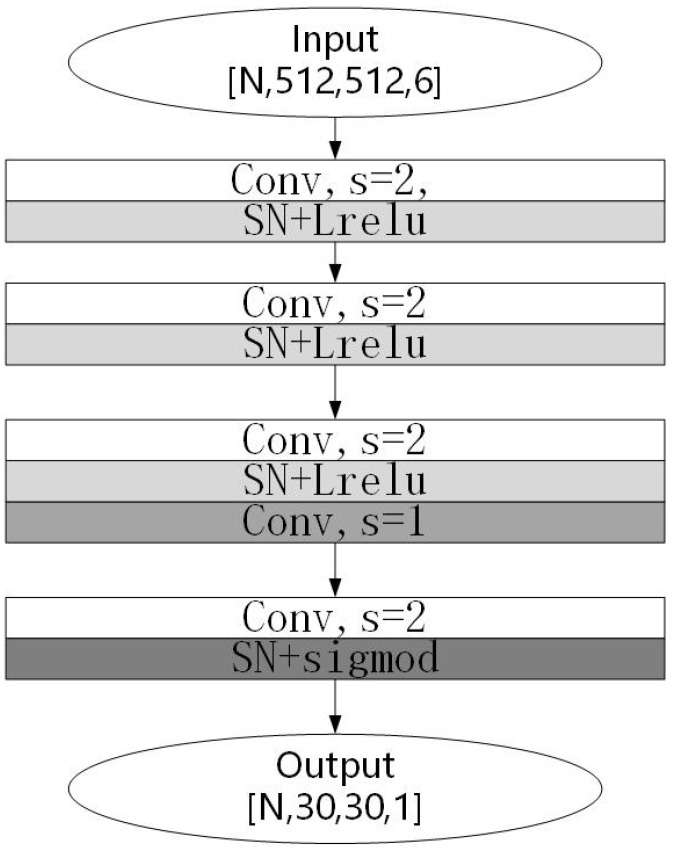
Discriminator network.

For the discriminator, this study made some targeted improvements while retaining the original discriminator structure, as shown in Figure 6. Firstly, spectral normalization was added. The training of GANs has been prone to pattern collapse or non-convergence, whereby the discriminator would then enter the ideal state early because it had a simpler structure than the generator and could not, therefore, provide effective gradient information to the generator. Based on this, Miyato [21] proposed the application of spectral normalization to the discriminator. Spectral normalization made the function conform to Lipschitz continuity, which could limit the drastic change in the loss function. The Lipschitz continuum was defined as follows:

In GAN, suppose the discriminator *D*: *I*→*R*, where *I* is the image space. If *D* is *K*-Lipchitz continuous, i.e., the maximum gradient of the function is *K*, then for any *x* and *y* in the image space, there is:

(1) ||D(x)−D(y)||≤K||x−y||

where ∥.∥ is L2-norm, and if *K* is taken to the minimum value, then *K* is called the Lipschitz constant. In this study, spectral normalization was used to replace the normalized layer of the discriminator’s convolutional kernel parameter matrix. The experiments showed that the proposed method could constrain the discriminator network in order to obtain stable training results.

Secondly, this paper used the Leaky Relu function as the activation function, which retained more image information than the Relu function.

## 5. Results and Analysis

In this study, the performance of the improved pix2pix was evaluated experimentally on a dataset of Go-game images. Firstly, the dataset was introduced, then the experimental environment and parameter settings of the model were provided, and finally, the evaluation index was used to measure the experimental results of the model.

### 5.1. Dataset

There were few publicly available datasets of Go images, so we considered constructing our own Go-game-image dataset. The steps of the dataset construction included data acquisition, data pre-processing, data annotation, and data-quality inspection. These four steps are described in detail below.

#### 5.1.1. Data Acquisition

**Figure 7:**
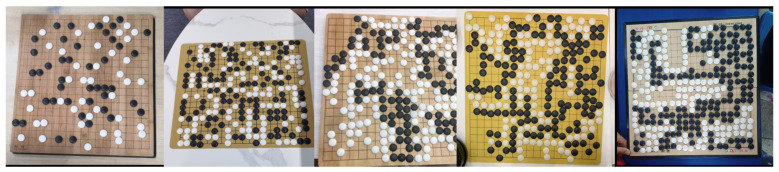
This is the original image, including different shooting angles, board materials, and background colors of the board image.

Data acquisition is the first step when building a dataset. We used the following devices: The camera type was a Canon EOS R5, the phone type was an Apple iPhone 13, and the tablet type was an iPad MK2N3CH/A. All the equipment was positioned directly and diagonally above the chessboard, and the linear distance from the board varied from 60 to 120 cm. The shooting conditions were in a bright indoor environment. The chessboard was made of wood, paper, and plastic, with a variety of background colors. Figure 7 shows some examples from the collected dataset, in which a total of 4500 Go images were collected.

#### 5.1.2. Data Pre-Processing

**Figure 8:**
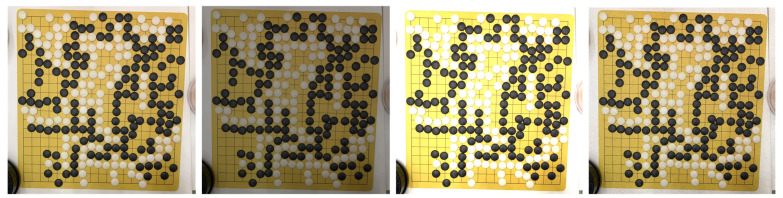
From left to right: original image, image with reduced brightness, image with increased brightness, and image with added noise.

The acquired images were filtered first, and the images with any occluded checkerboard area were removed. Then, in addition to the target board, there were extraneous details to remove in order to ensure that the board occupied the majority of the image. Finally, we carried out the data enhancement based on OpenCV, including a brightness adjustment and a noise addition. This was to simulate the recognition of the Go images under different lighting environments and noise levels in order to improve the robustness of the model to environmental factors. Figure 8 shows some examples of the data enhancement.

#### 5.1.3. Data Annotation

**Figure 9:**
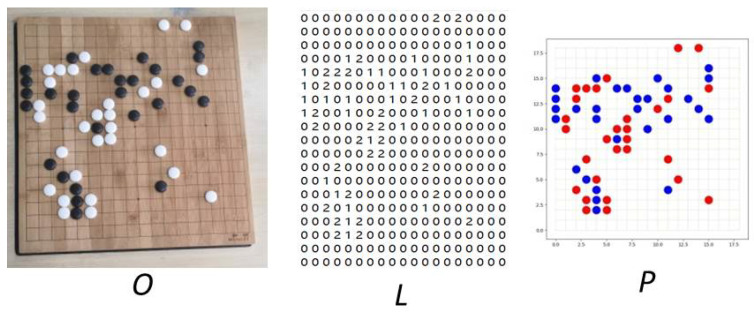
Image (*O*), label matrix (*L*), and label image (*P*).

Data annotation processes the data and converts it into information that can be recognized by a computer. Here, it was divided into two steps. Step one, the pre-processed image *O* was annotated to obtain a 19 × 19 label matrix *L*. Step two, the matrix *L* was drawn into the label image *P* by the MatplotLib, and the specific steps including drawing the canvas and coordinate axis, drawing the background grid, drawing the corresponding circle according to the matrix *L*, and finally, generating the recognizable chess-labeled image *P* (This is shown by *P* in Figure 9).The corresponding relationship among the original image, the label matrix *L*, and the label image *P* is shown in Table 1. Finally, *L* and *P* were combined to form a matched Go-image and labeled figure.

#### 5.1.4. Data-Quality Inspection

After the final label graph *P* was obtained, its quality needed to be evaluated to ensure the accuracy of the dataset. The rule of the quality inspection was to evaluate whether *O* and *P* were paired one to one. The quality inspection process was divided into two rounds: 100% coverage in the first round and 10% coverage in the second round. The two rounds of quality inspection could reduce the workload and improve the accuracy of annotation.

After the above steps, the dataset had a good standard for both quantity and quality. Finally, the images after the quality inspection were divided as follows: 80% of the images were selected as the training set (in the Supplementary Materials), and 20% of the images were selected as the test set (in the Supplementary Materials).

### 5.2. Experimental Environment and Parameter Settings

The experimental environment was as follows. The operating system was a CentOS Linux release 8.5.2111, the graphics card was a Tesla T4, and the processor was a Intel(R) Xeon(R) Platinum 8163 CPU @2.50GHz. The experiment was performed in python3.8, tensorflow1.7.1-cuda11.0 environment.

The parameters were set as follows. The generator learning rate was set to 0.0002, the discriminator learning rate was set to 0.00005, the batch training amount was set to 8, the number of training times was set to 200, and the other parameter settings were left as default. The Adam optimizer was used to accelerate the training process, and the input images were randomly flipped to improve the generalization of the model.

### 5.3. Evaluation Metrics

#### 5.3.1. Image Quality Evaluation Metrics

In this study, three indicators were used to evaluate the quality of the generated image, namely SSIM [22], PSNR [23], and FID [24]. SSIM is a structural similarity index measure, and it could quantify the structural similarity between the labels and the generated images; the score range was [0, 1], and the larger the value, the smaller the image distortion. PSNR is the peak signal-to-noise ratio, and it was mainly used to quantify the reconstruction quality of images and videos affected by lossy compression, where the higher the PSNR value, the better the image quality. FID is the Frechet inception distance score, and it is a measure to calculate the distance between the label and the feature vector of a generated image; where the lower the score, the more similar the two sets of images are or the more similar the statistics of the two. In the best case, the score of FID would be 0.0, indicating that the two sets of images were the same.

#### 5.3.2. Model Performance Evaluation Metrics

The ultimate goal of this study was to identify the chess pieces and their positions. These required high-quality generated images, as well as the color and the position of the red and blue circles in the images to be correct. We converted the generated images into a two-dimensional matrix *I* of 19 × 19 based on OpenCV. The specific steps were binary processing; contour retrieval; the drawing of the external rectangular frame and center point; and locating the red and blue circles. The corresponding relationship between the matrix *I* and the label matrix *L* in Table 1 were the same. For matrix *I*, its label *L* was evaluated from three aspects: accuracy, recall, and precision.

(2) $$Accuracy=\frac{TP+TN}{TP+FP+FN+TN}$$

(3) $$Recall=\frac{TP}{TP+FN}$$

(4) $$Precision=\frac{TP}{TP+FP}$$

where *TP* is true positive, *FP* is false positive, *FN* is false negative, and *TN* is true negative.

In addition, in order to pay more attention to the error rate of the chess-piece recognition, this study referred to [9] and added the following three evaluation indicators: *AERIP*, *AERI*, and *AERTD*. *AERIP* is the average error rate of erroneous intersection points, *AERI* is the average error rate of the images where errors appeared, and *AERID* is the average error rate of the images where errors could disrupt the judgment of the winner.

(5) $$AERIP=\frac{NEIP}{N\times 361}$$

(6) $$AERI=\frac{NIEP}{N}$$

(7) $$AERID=\frac{NIED}{N}$$

where *NEIP* is the number of erroneous intersection points, *NIEP* is the number of images where errors appeared, *NIED* is the number of images where errors could disrupt the judgment of the winner, and *N* is the total number of test images.

### 5.4. Ablation Experiment

**Figure 10:**
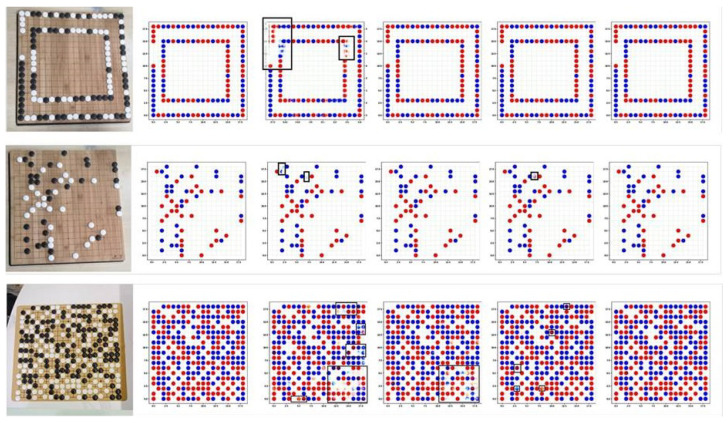
The results of ablation experiment on chess dataset. The columns, from left to right, include the original image, the label, Pix2pix, +DDC, +CCMA, and ours. The error results are marked by black boxes.

In order to verify the validity of each module in the algorithm, an ablation experiment was performed, as described in this section. The visualized results are shown in Figure 10. It was observed that the Go images generated by the original pix2pix model had unclear contours and image-noise points, and it was easy to distinguish between true and false images. After adding DDC, the image outline was obviously clear, indicating that the quality of the generated image had been effectively improved after adding the DDC module. However, for the target chess pieces, there were many position errors. After adding CCMA, the position of chess pieces was basically correct, which indicated that after adding CCMA module, the model paid attention to small target information. However, the chess pieces were the wrong color. Through the combination of the DDC and CCMA modules, the enhanced algorithm in this study had significantly improved the image quality. The generated images and labels were basically identical, and it was difficult to distinguish between true and false images.

The quantization results are shown in Table 2. It was observed that compared with pix2pix, the image quality of the model after adding DDC and CCMA was significantly improved, despite the parameter increase of 1.24M (million). It was reduced to an FID score of 0.8384, increased to an SSIM score of 0.9914, and increased to a PSNR score of 44.2977. For the recognition of the target chess pieces, the Accuracy, recall and precision of the proposed algorithm reached 0.9991, 0.9997, and 0.9982, respectively.

### 5.5. Comparison Experiment

In order to evaluate the performance of the proposed algorithm, it was compared with existing chess-image-recognition methods, including DenseNet, VGG-16, and Yolo v5. See Table 3 for comparison results. It was observed that compared with other methods, the algorithm proposed in this study had a higher accuracy of 0.9982, which was 2.61%, 1.01%, and 3.10% higher than the DenseNet, VGG-16, and Yolo v5 models, respectively. In addition, the algorithm in this study significantly reduced the average error rate (AERI) of the images with errors to 0.03, which was 11%, 6.5%, and 1.5% lower than DenseNet, VGG-16, and Yolo v5 models, respectively.

In addition, we quantitatively compared the parameter sizes (parameters in millions) and the inference speed (frames per second, FPS) of these models on the test set. The recognition method based on DenseNet actually contained two models, one for identifying the chessboard and the other for identifying the chess pieces. In the process of running the model, the chessboard and then the chess pieces needed to be identified first, so the FPS was reduced by half, as compared to the single model. The same was true for VGG-16. The recognition method based on Yolo v5 actually contained three models, a chessboard-recognition model and two chess-piece-recognition models, so it had the largest number of parameters and the lowest inference speed. The model in this study directly identified the whole board and its pieces, and after obtaining the label image *P*, the two-dimensional matrix *I* could be obtained based on OpenCV, and the chess pieces could be successfully identified. Although the number of parameters was larger than DenseNet and VGG-16, the inference speed was not much different because there was only one model. For Go-game image-recognition tasks, the error rate was a very important evaluation index, which was directly related to the final victory judgment. Therefore, the lower cost (slightly lower FPS) in exchange for a lower error rate was worth it.

**Figure 11:**
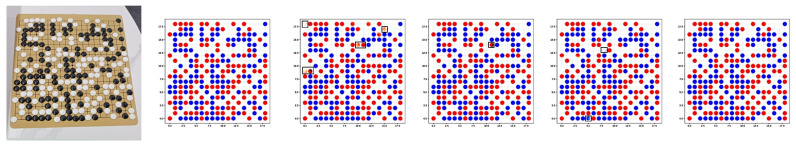
Results of the different attention mechanisms comparison experiment on the Go-image dataset. From left to right: original image, label, SENet, CBAM, CA, and CCMA. The error results are marked by black boxes.

In order to verify the effectiveness of the CCMA proposed in this study, SENet, CA, and CBAM were used in comparison experiments. The visualization results are shown in Figure 11. It was observed that the generation effect of SENet had the worst results, including missing pieces and unclear outlines. CBAM and CA were slightly better, but there were still situations where the outlines were not clear and there were noise points. Comparatively, the CCMA mechanism proposed in this study had the best generation effect, the outlines were clear, and the generated images were closest to the labeled sample.

The quantitative results are shown in Table 4. It was observed that the parameters of the CCMA model only increased by 0.08M, as compared to CA, but they decreased by 0.0542 in the FID index and increased by 0.6676 in the PSNR index. The effectiveness of the proposed CCMA mechanism was fully demonstrated.

## 6. Conclusions

In this study, an improved conditional-generation, adversarial-network Go-image-recognition algorithm was proposed. Through the introduction of a CCMA module, channel attention and coordinate attention were effectively combined, so the model could learn the feature information of the target that needed attention, that is, the information of the small target chess pieces. By introducing a DDC module, the dilated convolutions with different dilated rates were densely linked, so the model could learn long-distance contextual information at different scales. The experimental results showed that the improved method was superior to the existing methods, and the average accuracy of the chess-piece recognition was more than 99.99%. The experimental results on real Go-game images showed the effectiveness and the practicability of the proposed method. The research in this study also provides a potential reference for the recognition of small objects with few features. The dataset will be further enriched to improve the algorithm model.

## Figures and Tables

**Table 1:** The mapping between the image (*O*), label matrix (*L*), and label image (*P*).

Image (*O*)	Label Matrix (*L*)	Label Image (*P*)
Black chess	1	Blue circle
White chess	2	Red circle

**Table 2:** The results of the ablation experiment.

Method	Params (M)	FID	SSIM	PSNR	Accuracy	Recall	Precision
pix2pix	80.92	2.1952	0.9833	41.8361	0.9942	0.9949	0.9931
+DDC	81.37	0.9738	0.9892	41.7730	0.9982	0.9995	0.9977
+CCMA	82.16	0.8484	0.9906	43.0144	0.9997	0.9982	0.9967
ours	82.61	0.8384	0.9914	44.2977	0.9991	0.9997	0.9982

**Table 3:** The results of the comparison experiment.

Method	Accuracy	Recall	Precision	AERIP	AERI	AERID	Params (M)	FPS
DenseNet	0.9799	0.9699	0.9721	0.0037	0.1400	0.1050	67.63	68
VGG-16	0.9871	0.9852	0.9881	0.0022	0.0950	0.0700	60.05	73
Yolo v5	0.9985	0.9952	0.9672	0.0007	0.0450	0.0400	96.53	47
ours	0.9991	0.9997	0.9982	0.0004	0.0300	0.0150	82.61	66

**Table 4:** Results compared with other attention mechanisms.

Method	Params (M)	FID	SSIM	PSNR
SENet	81.42	1.4824	0.9894	39.6564
CBAM	83.54	0.9540	0.9899	41.7389
CA	82.08	0.9026	0.9902	42.3468
CCMA	82.16	0.8484	0.9906	43.0144

## Data Availability

Data is contained within the Supplementary Materials.

## Supplementary Materials

The dataset of this study can be downloaded at: https://github.com/zhengyanxia0310/dateset.git, The dataset contains two folders: the train set and the test set.
